# Tick-Borne Relapsing Fever Spirochetes in the Americas

**DOI:** 10.3390/vetsci3030016

**Published:** 2016-08-15

**Authors:** Job E. Lopez, Aparna Krishnavahjala, Melissa N. Garcia, Sergio Bermudez

**Affiliations:** 1 Department of Pediatrics, National School of Tropical Medicine, Baylor College of Medicine, Houston, 77030 TX, USA; Aparna.Krishnavahjala@bcm.edu (A.K.); mnolan@bcm.edu (M.N.G.); 2 Department of Molecular Virology and Microbiology, Baylor College of Medicine, Houston, 77030 TX, USA; 3 Departamento de Investigación en Entomología Médica, Instituto Conmemorativo Gorgas de Estudios de la Salud, P.O. Box 816-02593, City of Panama, Panama; bermudezsec@gmail.com

**Keywords:** relapsing fever spirochetes, *Borrelia*, *Ornithodoros*, argasid, ixodid

## Abstract

Relapsing fever spirochetes are tick- and louse-borne pathogens that primarily afflict those in impoverished countries. Historically the pathogens have had a significant impact on public health, yet currently they are often overlooked because of the nonspecific display of disease. In this review, we discuss aspects of relapsing fever (RF) spirochete pathogenesis including the: (1) clinical manifestation of disease; (2) ability to diagnose pathogen exposure; (3) the pathogen’s life cycle in the tick and mammal; and (4) ecological factors contributing to the maintenance of RF spirochetes in nature.

## 1. Introduction

Relapsing fever (RF) spirochetes are a significant cause of disease on five of seven continents, and are transmitted by argasid and ixodid ticks, and the human body louse. The pathogens are categorized as endemic (tick-borne) or epidemic (louse-borne), and all but two species (*Borrelia recurrentis* and *Borrelia duttonii*) are maintained in enzootic cycles with humans as accidental hosts [1,2]. In regions of Africa, the ecology and epidemiology of RF spirochetes have been extensively studied and the pathogens are a significant cause of child morbidity and mortality [3,4,5,6,7,8,9,10,11]. Outside of the African continent less is known regarding how RF spirochetes are maintained in nature.

This review primarily examines the ecology of tick-borne RF spirochetes in the Americas, with a focus on argasid-borne RF (ABRF). Moreover, since the epidemiology of ABRF in North America has been comprehensively reviewed [12,13,14,15] and little attention has been given to Latin America, in addition to the disease’s ecology our review also highlights epidemiological findings and case reports from Central and South America. We also review studies on *Borrelia miyamotoi*, an ixodid-borne RF (IBRF) species transmitted by *Ixodes* species, which was recently recognized to cause human disease [16]. While the last decade has resulted in a better understanding of how RF spirochetes are maintained in a tick-mammalian transmission cycle, there are deficiencies that remain and should be addressed. We conclude our review by addressing these critical questions and suggest actions suitable for progress in our understanding of ABRF and IBRF in the Americas.

## 2. Clinical Manifestation of Disease

In humans, ABRF presents with an onset of fever (104–107 °F) within four to 18 days after tick bite [17]. Acute disease is complemented with myalgia, headache, chills, diaphoresis, anorexia, nausea, and vomiting [14]. Febrile episodes may last three to four days, and are followed by an afebrile period of up to 10 days [14]. The cyclic nature of disease can continue for months if left untreated [17,18], and is due to antigenic variation [19]. An antibody response is generated against the predominant variable membrane protein (Vmp) produced on the surface of members within the spirochete population, resulting in pathogen clearance. However, the spirochetes switch to produce a Vmp variant that is not recognized by the host immune response, and a new population of spirochetes emerges in the blood [20,21].

Uncommon, yet severe, clinical manifestations of disease are associated with the systemic nature of the circulating ABRF spirochetes. Patients may develop acute respiratory distress, characterized by bilateral infiltrates and rales on chest X-rays [22]. Central nervous system involvement, including nuchal rigidity, facial paresis, vertigo, positive Kernig’s sign, and myocarditis has been noted [14]. Hepatosplenomegaly is palpable on physical examination, with an elevation of liver enzymes [14]. Cardiac involvement has been rarely reported, with electrocardiographic conduction delays and depression in ejection fraction on echocardiography being observed [23,24]. In the event of pregnancy, transplacental transmission can result in miscarriage [25].

RF spirochetes are susceptible to broad-spectrum antibiotics [14]. However, upon treatment 54% of ABRF patients had a Jarisch-Herxheimer reaction [12], which is characterized by a profound deterioration of symptoms including a sudden onset of fever, tachycardia and tachypnea, and blood pressure [26]. This pathophysiology results from a massive release of tumor necrosis factor by macrophages and is induced by spirochete surface lipoproteins [27].

As a recently recognized human pathogen, the clinical presentation of *B. miyamotoi* is less severe than ABRF. The spirochetes are neurotropic and can be detected in the cerebrospinal fluid of those displaying symptoms of meningoencephalitis [28]. Patients also present with headache, fever, chills, fatigue [29,30,31]. Although *B. miyamotoi* possess homologues for Vmps [32], it is unclear whether the pathogens undergo antigenic variation, and the number of relapses in the host is poorly understood.

## 3. Diagnosis of Exposure to RF Spirochetes

**Figure 1:**
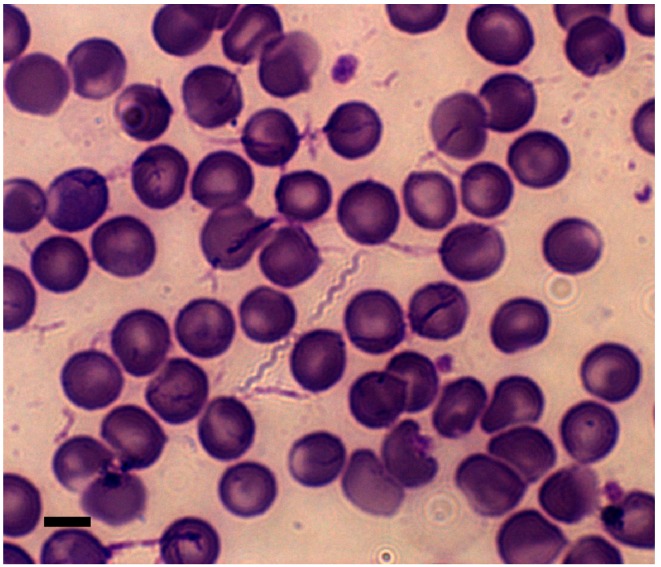
Giemsa-stained peripheral blood smear of a mouse infected by tick bite with *Borrelia turicatae*. The black bar represents 10 µm.

Currently there are no commercial diagnostic tests available for RF spirochetes, with national reference laboratories or academic laboratories providing detection capacities. Two primary methods of evaluating mammalian exposure are microscopy and molecular assays. RF spirochetes attain high densities in mammalian blood, at which point the pathogens can be visualized by dark field microscopy or Giemsa stained thin smears (Figure 1). While high bacterial loads in the blood are associated with fever, accurate diagnosis between febrile episodes is challenging because the pathogens are below the limit of detection [33]. During the course of infection as an antibody response is generated against RF spirochetes, molecular diagnostic assays are an alternative method to confirm mammalian exposure.

With the ecological overlap between RF and Lyme disease causing spirochetes, antigenic conservation between species, and observed serological cross-reactivity, identification of diagnostic antigens unique for a given disease group is important. The first diagnostic antigen discovered for RF spirochetes was glycerophosphodiester phosphodiesterase Q (GlpQ) [34]. A homologue of glpQ is absent from *B. burgdorferi* and the recombinant protein can discriminate between infections caused by RF and Lyme disease causing spirochetes [34]. Moreover, the protein may be used to diagnose early infection as IgM responses to recombinant GlpQ was detected in a cohort of infected patients from Ethiopia within four days after infection [35]. GlpQ also contains highly-conserved serologically cross-reactive epitopes between Old and New World species of RF *Borrelia* [36,37]. This is important when determining mammalian exposure in regions of the globe where it is unknown if RF spirochetes are circulating in nature.

A more recently discovered diagnostic antigen is the Borrelia immunogenic protein A (BipA). An immunoproteomic approach identified BipA as antigenic using serum samples from human patients and infected mice [35]. Similar to GlpQ, a BipA homologue is absent from *Leptospira* and Lyme disease-causing spirochetes [38]. BipA may also be a species specific antigen for RF spirochetes as the protein is highly divergent between species of RF spirochetes [39]. For example, serological responses from a canine and rodents experimentally infected by tick bite with *B. turicatae* failed to cross-react with recombinant BipA that originated from *B. hermsii* [39]. Currently, recombinant GlpQ and BipA offer the most thorough opportunity of accurate serodiagnosis of RF spirochetes. 

## 4. The Life Cycle of ABRF Spirochetes in the Mammal

Species of RF spirochetes circulate in an infectious cycle between the mammalian host and argasid or ixodid ticks. Salient differences between the biology of the two tick vectors are summarized in Table 1 [1,40,41], and ABRF and IBRF spirochetes have likely evolved unique mechanisms for vector colonization and mammalian infection. Currently, the life cycle of ABRF spirochetes within the mammal and tick is more defined than IBRF spirochetes (*B. miyamotoi*), and will be further reviewed.

**Figure 2:**
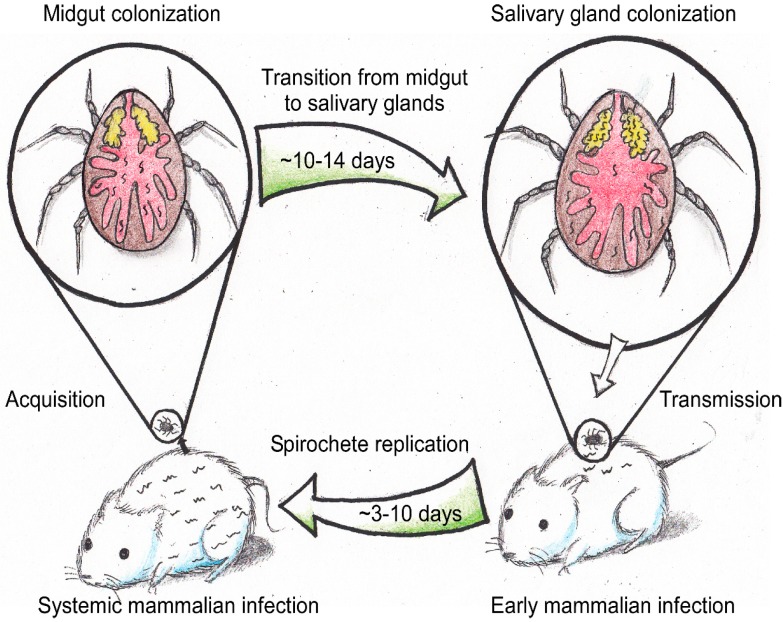
The tick-mammalian transmission cycle of ABRF spirochetes. In the tick, the salivary gland population of RF spirochetes is essential for mammalian infection because of the rapid feeding behavior of the tick. Entry into the mammal is characterized by early infection, and the pathogens are likely preadapted to evade innate immunity. During the following three to 10 days, RF spirochetes subvert the host antibody response leading to systemic infections. This phase of the pathogen’s life cycle is characterized by evasion of the host antibody response through antigenic variation, and replication to densities upwards of 1 × 10^7^ spirochetes per milliliter of blood. During an acquisition bloodmeal, RF spirochetes enter and colonize the midgut. Within 10–14 days a population exits the midgut and migrates to colonize the salivary glands, completing the life cycle of the spirochetes in the argasid tick vector.

Transmission studies indicate that ABRF spirochetes are likely preadapted in the tick for mammalian entry. While the infectious dose delivered through the tick saliva remains unknown, the observed rapid transmission of *Borrelia turicatae* within 15 s of tick bite [42] indicates that the subsets of spirochetes preadapted to survive innate immunity will continue their life cycle in the mammal. For example, work by Woodman and Alugupalli demonstrated the importance of macrophages, dendritic cells, and B1 B cells (innate-like B cells) in controlling RF spirochete infection [43,44]. However, during the first three to five days after transmission ABRF spirochetes also replicate. Therefore, early infection is characterized by an interplay between innate immunity and spirochete propagation (Figure 2).

The transition from early infection to systemic infection (Figure 2) is characterized by the upregulation of the expression locus involved with antigenic variation [45]. The pathogens replicate to high densities in the blood, and infection is cyclic with antigenic variation as the driving force to ensure the spirochetes continue their life cycle [19,46]. For example, the inability of *Ornithodoros hermsi* to become colonized by *Borrelia hermsii* while feeding on an infected animal between relapses indicated the significance of antigenic variation in providing multiple opportunities for vector acquisition [47].

The number of spirochete relapses in a competent mammalian host was recently modeled in the *O. hermsi-B. hermsii* system to predict factors required to keep a community endemic [48]. Johnson and colleagues conducted field studies in a unique ecological setting on Wild Horse Island, Flathead Lake, Lake County, MT, USA. The island is endemic with *B. hermsii* and exclusively inhabited by deer mice (*Peromyscus maniculatus*) and pine squirrels (*Tamiasciurus hudsonicus*) [48,49]. These factors provided an opportunity to develop a single and coupled host-vector model to determine the number of relapses predicted for the island to remain endemic, with R_0_ > 1 indicating endemicity and R_0_ < 1 is a disease-free equilibrium. In the single host system where pine squirrels are known to maintain *B. hermsii*, R_0_ > 1 was observed at four relapses. Interestingly, in a coupled host-vector system that included incompetent deer mouse, seven relapses within pine squirrels were predicted to be required in order to produce an R_0_ > 1. These studies established a framework for understanding additional RF spirochete systems, and demonstrate the importance of defining the dynamics of mammalian host competency with keeping a community endemic. 

## 5. The Life Cycle of ABRF Spirochetes in the Tick Vector

During an infectious bloodmeal, RF spirochetes enter the midgut (Figure 2). Although the precise infectious dose required for *Ornithodoros* colonization remains unclear, studies with the *B. hermsii-O. hermsi* model suggested that as few as 30 spirochetes were sufficient to successfully infect ticks [33,47]. Cohorts of second nymphal *O. hermsi* were fed for 13 consecutive days on an infected mouse, which spanned two spirochetemic episodes [47]. Estimating the bloodmeal volume for second stage nymphs indicated that they imbibed ~30 spirochetes [33,47], with 50% of the ticks becoming colonized by *B. hermsii* [47].

After feeding, the midgut serves as the first site of RF spirochete adaptation. Nakajima and coworkers reported that during the bloodmeal the antimicrobial peptides, defensin A and B, were upregulated in *Ornithodoros moubata* [50,51,52], suggesting that RF spirochetes likely evolved mechanisms to subvert vector immunity. In the following 10 to 14 days after feeding, a population of spirochetes exit the midgut and begin to colonize salivary glands (Figure 2). During migration through the hemocoel and within the salivary glands, ABRF spirochetes continue to face immunological pressures [50,53,54]. Transcriptional and proteomic studies of *O. parkeri* identified the production antimicrobial peptides in the salivary glands [54], and indicated that the tissues are another environment exerting immunological pressures on RF spirochetes. Thus, in a persistently-infected tick two populations must adapt to vector immunity, those in the midgut and others in the salivary glands [55].

ABRF spirochete transmission occurs within seconds of tick bite [42,56], and the salivary gland population is essential to continue the spirochetes’ life cycle in the mammal. The rapidity of ABRF spirochete transmission indicates that the pathogens are preadapted for mammalian entry [42,46]. Raffel and colleagues demonstrated this by deleting the *B. hermsii* variable tick protein, which the spirochetes predominantly produce in the tick salivary glands [45]. Inactivating the gene resulted in a noninfectious phenotype after tick bite [46]. Interestingly, the mutant’s ability to colonize *O. hermsi* salivary glands demonstrated the importance of the protein during early mammalian infection.

Transcriptional assessment of *B. turicatae* indicated that large linear megaplasmids of RF spirochetes likely play important roles in the vector and preadapting the pathogens for mammalian entry [57]. Over 60% of open reading frames (ORFs) on the megaplasmid were upregulated during in vitro cultivation at a temperature mimicking the tick environment. A cluster of ORFs localized toward the 3’ end was further evaluated in cohorts of *O. turicata*, which confirmed the genes’ upregulation in the tick [57]. As these proteins are characterized, subsets will likely be identified that are important for midgut and salivary gland colonization, while others may preadapt the spirochetes to evade the selective pressures encountered during early mammalian infection.

A defining characteristic of ABRF spirochete-*Ornithodoros* interactions is vector specificity [1,58,59]. For example, *O. hermsi, O. parkeri*, and *O. turicata* transmit *B. hermsii, B. parkeri*, and *B. turicatae*, respectively. While the mechanism is still unknown, the salivary glands may be the restricted environment. Work by Schwan demonstrated that *B. hermsii* colonized and disseminated from the midgut of *O. hermsi*, *O. parkeri*, and *O. turicata*; however, only *O. hermsii* subsequently transmitted the pathogens to mice [60]. As the field of transcriptomics has emerged, the determination of salivary gland gene expression from *Ornithodoros* species is feasible, and defining the intricacies of vector colonization is promising.

## 6. Ecology of ABRF in North America

In North America, the *Ornithodoros* vectors for ABRF spirochetes are distributed in endemic foci in Western Canada and the United States, across the southern portion of the country into Mexico [40]. There are four likely argasid tick vectors of RF spirochetes that cause human disease, *Ornithodoros parkeri*, *Ornithodoros hermsi*, *Ornithodoros turicata*, and *Ornithodoros talaje*, which transmit *B.* *parkeri*, *B. hermsii*, *B. turicatae*, and *B. mazzottii*, respectively. *Ixodes scapularis* and *Ixodes pacificus* transmit *B. miyamotoi*, the species of IBRF spirochetes, and the ticks are found in the Western and Northeastern United States [61,62,63]. Since the ecology of *Ixodes* species has been well described [64], and there is a paucity of information regarding reservoir host competency for *B. miyamotoi*, we focus the remainder of this review on the ecology of ABRF spirochetes and their vectors.

### 6.1. O. parkeri-B. parkeri

**Figure 3:**
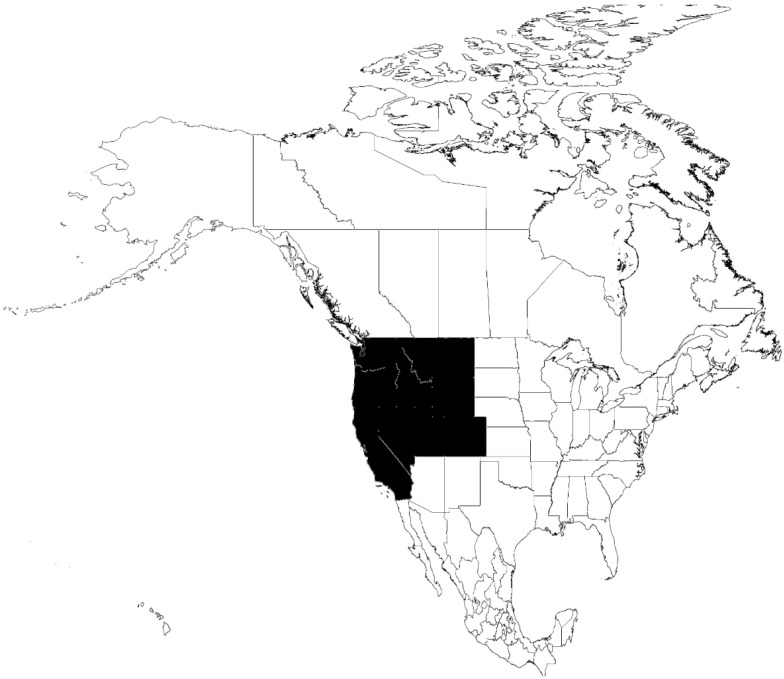
Distribution of *Ornithodoros parkeri* in North America.

Currently, a human isolate does not exist for *B. parkeri*, yet the species has been implicated as a possible cause of RF because of tick collections from locations of suspected human exposure [65,66]. These studies date back to 1934, when ticks were obtained within burrows from semi-arid locations at elevations at sea level to over 2000 m [65,66,67]. Burrows were identified throughout the Western United States (Figure 3) in sagebrush and grassy slopes with primary inhabitants of prairie dogs, rabbits, rodents, and owls [66,67]. The ticks are nonselective feeders, engorging off of man, white mice, rats, guinea pigs, and nonhuman primates [66,67]. Furthermore, work by Davis initially reported cannibalism between *Ornithodoros* ticks, when he observed unfed *O. parkeri* feeding on engorged ticks, which left the host tick unaffected [66]. These studies suggest that cohorts of fed ticks could continue the life cycle of RF spirochetes within a burrow or nest community by becoming a bloodmeal source to unfed ticks.

### 6.2. O. hermsi-B. hermsii

**Figure 4:**
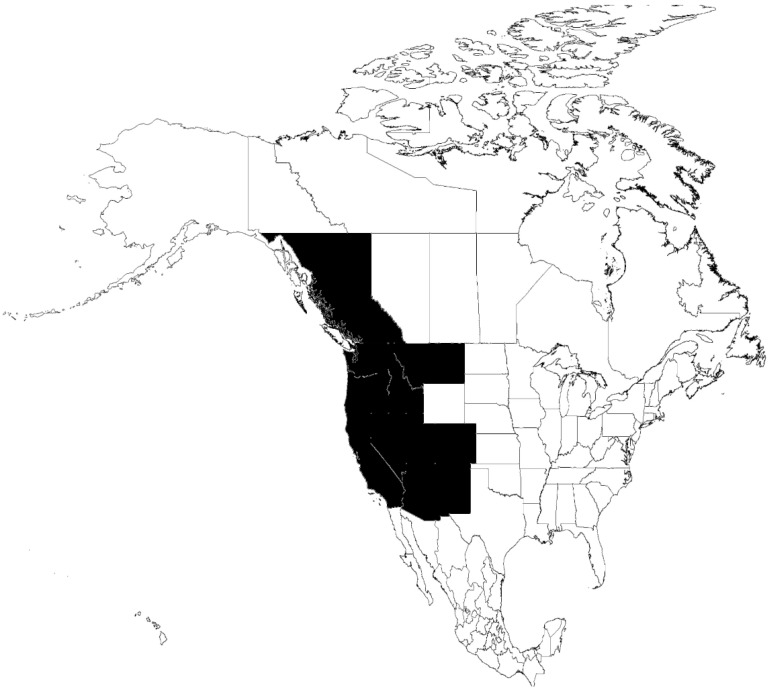
Distribution of *Ornithodoros hermsi* in North America.

The ecology of *B. hermsii* and the tick vector is the most studied and defined of the RF spirochete species in the Americas [48,68,69,70]. Throughout the Western United States and Southern British Columbia *O. hermsi* is distributed above elevations of 900 m [13,40,71] (Figure 4). The spirochetes circulate in enzootic cycles between ticks and rodents, with evidence that woodrats (*Neotoma* spp.), deer mice (*Peromyscus* spp.), chipmunks (*Tamias* spp.), and pine squirrels (*Tamiasciurus* spp.) are suitable hosts to varying degrees [48,49,68,70,72,73,74].

Seminal work by Burgdorfer et al. first evaluated vertebrate host competency to *B. hermsii* [73]. Infection studies by needle inoculation or tick bite were conducted on chipmunks, pine squirrels, flying squirrels, Columbian ground squirrels, golden-mantled ground squirrels, wood rats, white-footed deer mice, and meadow voles. Of these small mammals, *B. hermsii* only infected pine squirrels, chipmunks, and meadow voles, as determined by microscopic evaluation of blood. However, more recent serological surveillance studies indicated that *B. hermsii* also propagates in deer mice throughout California [74], indicating that these small mammals are competent reservoirs. Moreover, the study was reported in 1970, and a year later culture medium was developed, to isolate *B. hermsii* [75]. Consequently, few spirochete isolates existed and it was unknown at the time that *B. hermsii* separated into two genomic groupings (GGI and GGII) [76]. Repeating the rodent infection studies with GGI and GGII isolates of *B. hermsii* may expand our understanding on host competency between genomic groups and further clarify the maintenance of *B. hermsii* in nature.

Evidence is also mounting that migratory birds and large vertebrates are involved in the ecology and dispersal of *B. hermsii*. The extraction and typing of *B. hermsii* DNA from the liver of a deceased northern spotted owl suggested that the spirochetes could establish an infection in birds [77,78]. Subsequent identification of identical genotypes of *B. hermsii* isolated from human patients from Lake Co., Montana and Siskiyou Co., California further indicated a role of migratory animals in spirochete dispersal [76]. The study also reported that chickens and quail support all life cycles of *O. hermsi*, and that *B. hermsii* was able to cause spirochetemia after needle inoculating chickens [76]. The ecology of *B. hermsii* is becoming defined in a forest landscape with terrestrial (chipmunks) and arboreal (squirrels and birds) vertebrates that maintain the pathogens, and squirrels may serve as a bridge for introducing *B. hermsii* and the vector into migratory bird habitats. 

Further evidence that the ecology of *B. hermsii* may be more complex than a rodent-tick infectious cycle comes from the isolation of the species from a dog and surveillance studies in mule deer [69,79]. *B. hermsii* was isolated from the blood of a dog in Washington State, which suggests that wild canids may maintain the pathogens [79]. Furthermore, *B. hermsii* DNA was detected in the blood and lymph nodes of mule deer across Nevada, with seven percent of the animals positive [69]. While *O. hermsi* is associated within rodent and possibly bird nests [40], Nieto and colleagues pose an interesting scenario for the transmission of *B. hermsii* by *Ornithodros coriaceus*, a species primarily identified within leaf litter and deer beds [40]. More studies are warranted to further understand this vector-*Borrelia* species connection.

### 6.3. O. turicata-B. turicatae

**Figure 5:**
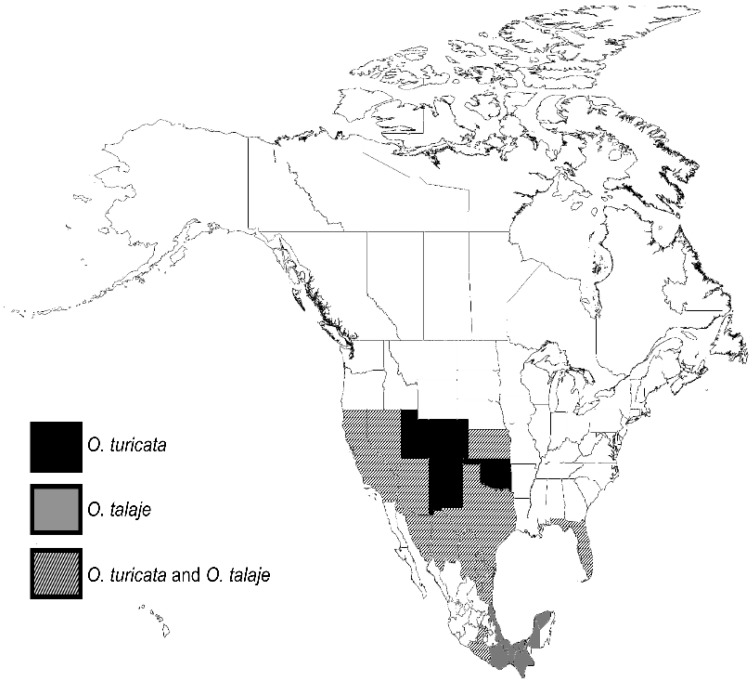
Distribution of *Ornithodoros turicata* and *Ornithodoros talaje* in North America.

In the Southern United States and Northern Mexico, *O. turicata* is likely the primary vector of RF spirochetes (Figure 5). Historic reports have described the ticks into Central and South America [80], yet a current understanding of the tick’s distribution remains vague. In the United States, ecological studies of *O. turicata* have described a western and eastern population of tick, with a geographical gap comprised of Louisiana, Mississippi, and Alabama. *O. turicata* has consistently been collected from gopher tortoise dens in Florida [81,82,83], while similar field studies throughout Mississippi analyzing arthropod communities of dens failed to recover the ticks [84].

The geographical gap between Texas and Florida may be explained by current climate conditions. Utilizing a maximum entropy species distribution model to predict regions where *O. turicata* circulate, Dondalson and colleagues identified environmental variables that may explain the absence of *O. turicata* in Louisiana, Mississippi, and Alabama [81]. Low temperatures during the wettest quarter of the year, high temperatures during the driest quarter, and the amount of precipitation in the region during the driest quarter may produce an environment that would not facilitate the establishment of the ticks.

With the two isolated populations of *O. turicata*, the eastern population has been considered a subspecies [85]. Beck and coworkers proposed that the biological differences and geographic separation warranted the eastern population being designated *O. turicata Americanus* [85]. Determining vector competency of eastern and western *O. turicata* populations with isolates of *B. turicatae* obtained throughout the southern United States is an important step toward understanding pathogen emergence across geographical distances.

Vertebrate host competency for *B. turicatae* is an additional aspect of infectious disease ecology needing clarity because of the nonselective feeding behavior of *O. turicata*. Ticks have been collected from a variety of habitats including rodent and burrowing owl nests, coyote and reptile dens, and within caves that are inhabited by a variety of bloodmeal sources [40,81,82,83]. Two likely hosts include rodents and wild canids. Rodents are known hosts for nearly all species of RF spirochete [10,68,72,86,87], and the animals are susceptible to *B. turicatae* infection by tick bite [39,42]. Evidence for a role of wild canids in the maintenance of *B. turicatae* comes from the recovery of the species from domestic dogs in Texas and Florida, and laboratory transmission studies with *O. turicata* [39,88,89].

The biology of *O. turicata* and dynamics between the vector and *B. turicatae* facilitate the emergence of the tick and pathogen. *O. turicata* are promiscuous feeders [40,85,90], and the vector is able to endure at least five years of starvation during which *B. turicatae* remains infectious upon subsequent feeding [90]. The ticks also maintain *B. turicatae* transovarially, as first demonstrated by work from Francis after his accidental exposure to two larvae [90]. The resilience of *O. turicata*, nonselective feeding behavior of the tick, and life cycle of *B. turicatae* within the vector indicates that the parameters are in place for the emergence of this species of ABRF spirochete.

### 6.4. O. talaje-B. mazzottii

*O. talaje* is an understudied vector of RF spirochetes in North, Central, and South America [40] (Figure 5). Transmission studies to humans by Panamanian *O. talaje* (further detailed below) indicated that the tick is a competent vector of the pathogens [91]. In North America, work by Mazzotti reported the first recovery of a RF spirochete from *O. talaje* in 1953 from Mexico, and he subsequently sent Dr. Gordon Davis infected ticks to further evaluate [59]. The spirochete was designated *Borrelia mazzottii* in honor of the researcher’s contribution to the field of RF spirochetes, and the following studies demonstrated vector specificity of this *Borrelia* strain. *O. talaje* recovered from Mexico, Guatemala, and Panama, *Ornithodoros puertoricensis* and *Ornithodoros rudis* collected in Panama, Colombia, and Ecuador, and *Ornithdoros dugesi*, *Ornithodoros nicollei*, and *O. turicata* from Mexico were infected with *B. mazzottii*. Upon subsequent tick feedings, only *O. talaje* from Mexico and Guatemala transmitted the spirochete to mice. The remaining “nontransmitters” were triturated and injected into mice, of which none became infected. This work suggested that *B. mazzottii* failed to colonize the “nontransmitting” ticks.

Gordon’s transmission studies with *O. talaje* that originated from North, Central, and South America indicated the complexity of soft tick systematics from this region of the globe. At the nymphal and adult stages, *O. talaje* is virtually indistinguishable from other closely-related species, such as *O. puertoricensis* [92]. Moreover, the phylogenetic classification of argasids that was established by Hoogstraal was based on morphological and biological characteristics, behavior, and tick development [93]. Therefore, the species specificity of *B. mazzottii* for *O. talaje* collected in Mexico and Guatemala suggests that the biological differences in vectorial competency may separate the tick species from those obtained in Central and South American. Furthermore, since the studies were also conducted with nymphs and adults, it is conceivable that *O. talaje* collected in Panama may have been *O. puertoricensis* [59], which is also distributed in the country [94]. Clearly, genetic information from *O. talaje* obtained throughout the Americas is needed and will clarify ambiguities with the vectors distribution.

**Figure 6:**
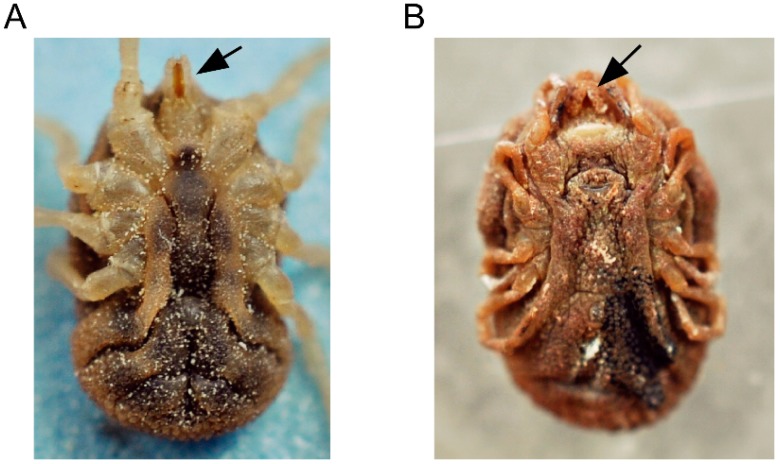
Morphological characteristics between *Ornithodoros turicata* and *Ornithodoros talaje*. *O. turicata* (**A**) and *O. talaje* (**B**) that were collected from a burrowing owl nest in Southern Texas. The mouthparts of *O. turicata* are exposed (**A**, black arrow) while “cheeks” cover the mouthparts of *O. talaje* (**B**, black arrow).

An interesting ecological finding of *O. talaje* is the collection of the ticks in identical niches as *O. turicata*. Our ongoing field studies in Southern and Central Texas continue to recover *O. talaje* in the same woodrat and burrowing owl nests as *O. turicata*. As previously stated, nymphal and adult *Ornithodoros* ticks can be challenging to specieate [40]. However, keying the collected ticks revealed that two species were recovered [40], with *O. talaje* adults discernable from *O. turicata* with the presence of cheeks covering the mouthparts and separation of the first and second coxae (Figure 6).

The feeding behavior of *O. talaje* larvae provides ample opportunity for the vector’s dispersal. Larvae are long-term feeders that remain attached to the vertebrate host for up to five days, while subsequent nymphs and adult *O. talaje* engorge rapidly [95]. As research attention is focused toward *O. talaje*, defining the dispersal and public health burden of this understudied tick and pathogen will be possible.

## 7. ABRF in Central America

Panama had some of the earliest accounts of RF in the Americas. Observations from the country at the turn of the 20th century paralleled those of Dutton and Todd, who first demonstrated that spirochetes were tick-borne pathogens that caused human disease in Africa [96]. For example, in 1907 Darling reported the detection of spirochetes in blood examinations of patients admitted to Commission hospitals in the Canal Zone [97]. During a 26-year period 117 cases were diagnosed [98]. However, as noted by Dunn and Clark, these patients were among employees of the Panama Canal who could afford health care, and the disease burden among the impoverished was unknown [98].

By 1921 it became clear that RF was a tick-borne disease. Bates and colleagues described several cases of RF in young men who were hunting in the Arraiján district of Panama [91]. The hunters “showed marks of many insect bites” and an investigation of their sleeping quarters resulted in the collection of *O. talaje* from the men’s bamboo-constructed beds. In a series of experiments, rats and nonhuman primates were infected with triturated ticks and by bite, respectively. To conclusively confirm that RF spirochetes caused disease and *O. talaje* was the vector, human volunteers were inoculated with infected rat blood or by tick bite and disease progression confirmed [91].

Field studies in 1933 from the Gorgas Memorial Laboratory provided clues into the ecology of RF spirochetes in Panama [98]. RF spirochetes were detected in the blood of a variety of mammals, including calves and horses. While it was unclear whether these large mammals maintain RF spirochetes in nature, detection of spirochetes in squirrel monkeys, opossums, and armadillos was more revealing in the pathogen’s ecology [98]. Sixty-one wild-caught opossums were evaluated, and spirochetes were detected in the blood of ~10%. In a small cohort of 21 nine-banded armadillos, two animals had active infections. To determine whether the animals were susceptible to a human isolate of RF spirochete obtained in Panama, two clean armadillos were needle inoculated with infected blood from the patient. One animal became highly spirochetemic by the second day after inoculation and succumbed to infection within nine days. The second armadillo maintained a prolonged cyclic infection for a month and then euthanized. These studies were the first to implicate opossums and armadillos in the ecology of RF spirochetes.

**Figure 7:**
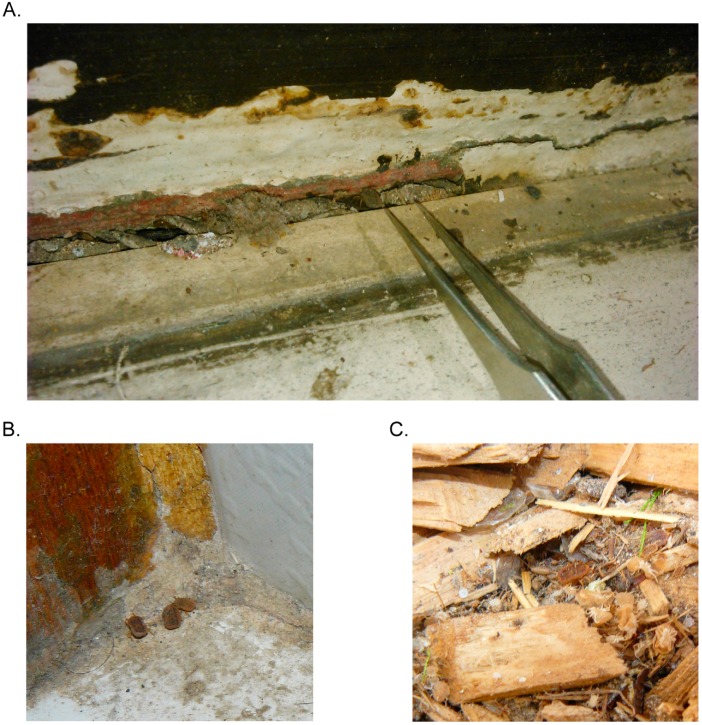
Adult *Ornithodoros puertoricensis* collected in a human dwelling in Ancon, Panama City, Panama (**A**,**B**); Immature and adult *O. puertoricensis* in a reptile terrarium in Escobar, Colon, Panama (**C**).

While RF spirochetes and their vectors have been neglected in Central America, evidence exists that the pathogens remain a public health problem. A recent case report of a tourist traveling along the Belize-Guatemala border indicated that the RF spirochetes continue to circulate in northern Central America [99]. Moreover, ongoing studies in Panama continue to identify *O. puertoricensis,* a putative vector, in domestic settings (Figure 7) and throughout the country [94,100,101,102]. The ecological work from Panama in the 1930s established the framework for current studies to understand RF spirochete maintenance in nature and disease burden on humans.

## 8. ABRF in South America

Similar to Central America, there is little information regarding tick-borne RF in South America; however, evidence from Brazil and Bolivia indicate that the pathogens remain endemic. *Ornithodoros brasiliensis*, locally known as the “mouro” tick, was an implicated vector for RF spirochetes as early as 1931 after patients displayed headache, dyspnea, and fever after tick bites [103]. Through a collaborative effort between de *B. Aragão*, di Primio and Davis, at the Instituto Oswaldo Crus, Rio de Janeiro, Brazil and the Rocky Mountain Laboratory, Hamilton, MT, USA, transmission studies were described with ticks [104]. Feeding *O. brasiliensis* collected from human dwellings on rodents resulted with transmission of spirochetes, with guinea pigs becoming febrile. With observed specificity between RF spirochetes and a given tick vector, Davis proposed that the bacteria be named *Borrelia brasiliensis* [104]. The subsequent half century resulted in an absence of reports of the tick, and it was thought that *O. brasiliensis* was potentially eradicated or extinct [105].

In 2011, Martins and colleagues reported the public health concern of *O. brasiliensis*, as the bite was associated with an intense systemic reaction resulting in hospital admissions [106]. Moreover, the patient described the death of a pet that was parasitized by the ticks [106]. The ticks are aggressive toward humans and animals, and have been collected in domestic and peridomestic settings of the Southern Brazilian highlands above 900 m [103,105,107]. *O. brasiliensis* buries itself in ~5–40 mm of aluminic humic cambisol acidic soil under human dwellings, sheds, and storehouses [105]. In addition to a potential vector of RF spirochetes, the bite of *O. brasiliensis* is associated with necrosis of the attachment site and delayed wound healing [107,108,109]. More work is needed to understand the ecology and public health significance of *O. brasiliensis*.

Evidence also exists for RF in Bolivia [110,111]. Ciceroni and colleagues reported that Guarani Indians and mestizos from Camiri, Boyuibe, and Gutierrez, Bolivia had detectable serological responses to *B. turicatae* and *B. parkeri* by indirect immunofluorescent assay after adsorption to *Treponema phagedenis* [111]. Eliminating cross-reactive antibodies between RF- and syphilis-causing spirochetes was important; however, these findings do not rule out the possibility that the patients were exposed to Lyme-causing *Borrelia*. Furthermore, Parola and coworkers reported the detection of *Borrelia* DNA in *Ornithodoros* ticks [110]. The ticks were collected from rocky outcrops in the Cochabama Department, Bolivia, at an elevation of 2500 m. While morphological evaluation of the ticks grouped them as *O. talaje*, molecular information is absent for this species, and the collected ticks may be closer related to a new species*, Ornithodoros rioplatensis* [92].

## 9. Conclusions and Future Directions

RF spirochetes are primarily transmitted by argasid ticks in the genus *Ornithodoros*, while *B. miyamotoi* is now emerging as a human pathogen transmitted by *Ixodes* species. With the recent ability to culture *B. miyamotoi* [112], it is now possible to understand the intricacies of vector colonization and transmission. For example, given that the tick is a long-term feeder, does *B.* *miyamotoi* persistently colonize the salivary glands of *Ixodes* species? Alternatively, do the spirochetes predominantly reside in the midgut and then migrate to the salivary glands during tick feeding, as observed for *Borrelia burgdorferi*, and how do co-infections with Lyme disease-causing spirochetes affect vector competency? With recent attention on *B. miyamotoi* as a human pathogen, it is likely that the next decade will result in a better understanding of the pathogen’s life cycle in the vector.

Important aspects of ABRF spirochete pathogenesis that should be considered are a better understanding of tick and vertebrate host competency, and how it relates to vector and pathogen dispersal throughout the Americas. Furthermore, the disease burden of RF spirochetes in North, Central, and South America is either unknown or likely under reported, yet the tick vector continues to be identified in domestic and peridomestic settings. The ability to accurately serodiagnose exposure to the pathogens will facilitate ecological and epidemiological studies to better understand RF spirochete circulation amongst at-risk populations.

## Figures and Tables

**Table 1:** Biological differences between *Ixodes* and *Ornithodros* species.

Biological Traits	*Ixodes* spp.	*Ornithodoros* spp.
Life span	2–3 years	5–20 years
Nymphal stages	1	>7
Feeding strategy	Questing/Nidicolous **^A^**	Nidicolous
Feeding duration	5–7 days	5–60 min
Transmission	unknown	~15 s **^B^**

**^A^** Nidicolous-cavity, nest, or den dwelling; **^B^** Transmission of *Borrelia turicatae.*
