# Erratum: Lopez, J.E., et al. Tick-Borne Relapsing Fever Spirochetes in the Americas. *Vet. Sci.* 2016, *3*, 16

**DOI:** 10.3390/vetsci6040098

**Published:** 2019-12-04

**Authors:** Job E. Lopez, Aparna Krishnavajhala, Melissa N. Garcia, Sergio Bermudez

**Affiliations:** 1 Department of Pediatrics, National School of Tropical Medicine, Baylor College of Medicine, Houston, TX 77030, USA; Aparna.Krishnavahjala@bcm.edu (A.K.); mnolan@bcm.edu (M.N.G.); 2 Department of Molecular Virology and Microbiology, Baylor College of Medicine, Houston, TX 77030, USA; 3 Departamento de Investigación en Entomología Médica, Instituto Conmemorativo Gorgas de Estudios de la Salud, City of Panama PO Box 816-02593, Panama; bermudezsec@gmail.com

The authors wish to make the following correction to this paper [1]: The author name “Aparna Krishnavahjala” should be “Aparna Krishnavajhala”.

We apologize for any inconvenience caused to the readers.
